# Microcystin-LR Degradation and Gene Regulation of Microcystin-Degrading *Novosphingobium* sp. THN1 at Different Carbon Concentrations

**DOI:** 10.3389/fmicb.2019.01750

**Published:** 2019-08-06

**Authors:** Juanping Wang, Chang Wang, Qi Li, Mengyuan Shen, Peng Bai, Jionghui Li, Yan Lin, Nanqin Gan, Tao Li, Jindong Zhao

**Affiliations:** ^1^State Key Laboratory of Freshwater Ecology and Biotechnology, Institute of Hydrobiology, Chinese Academy of Sciences, Wuhan, China; ^2^University of Chinese Academy of Sciences, Beijing, China; ^3^Key Laboratory of Combinatorial Biosynthesis and Drug Discovery, Ministry of Education, School of Pharmaceutical Sciences, Zhongnan Hospital, Wuhan University, Wuhan, China; ^4^State Key Laboratory of Protein and Plant Genetic Engineering, College of Life Sciences, Peking University, Beijing, China

**Keywords:** microcystin-LR, biodegradation, *Novosphingobium* sp. THN1, carbon availability, gene expression, transcriptome

## Abstract

The bacterium *Novosphingobium* sp. THN1 (THN1) is capable of degrading microcystin-LR (MC-LR). To study the ability of THN1 to degrade MC-LR and its possible mechanism(s) of regulation, we analyzed the effect of carbon concentrations on the degradation process. The MC-LR degradation rate peaked early and then declined during MC-LR biodegradation. Decreased levels of carbon in the medium caused the degradation peak to occur earlier. The expression of the functional gene *mlrA*, encoding a microcystinase, showed a similar trend to the MC-LR degradation rate at various carbon concentrations (r^2^ = 0.717, *p* < 0.05), suggesting that regulation of *mlrA* expression may play an important role in MC-LR degradation by THN1. The total bacterial biomass decreased when the carbon source was limited and did not correlate with the MC-LR degradation rate. Transcriptomic analysis showed that MC-LR degradation differentially regulated 62.16% (2597/4178) of THN1 genes. A considerable number of differentially expressed genes (DEGs) during MC-LR degradation encoded proteins related to carbon-, nitrogen-, and amino acid-related pathways. At 2 h of MC-LR degradation, most DEGs (29/33) involved in carbon and nitrogen metabolism were downregulated. This indicated that MC-LR may regulate carbon and nitrogen pathways of *Novosphingobium* sp. THN1. KEGG pathway analysis indicated that the upregulated DEGs during MC-LR degradation were mainly related to amino acid degradation and substrate metabolism pathways. Particularly, we detected increased expression of glutathione metabolism-related genes from transcriptomic data at 2 h of MC-LR degradation compared with the gene expression of 0 h, such as GST family protein, glutathione peroxidase, S-(hydroxymethyl) glutathione dehydrogenase, and glutathione-dependent disulfide-bond oxidoreductase that have been reported to be involved in microcystin degradation.

## Introduction

Microcystins (MCs) are a family of monocyclic heptapeptides having a general structure composed of five D-amino acids and two variable L-amino acids. They are synthesized by multienzyme complexes encoded by the *mcy* gene cluster and produced by some genera of cyanobacteria, including *Microcysits, Anabaena, Planktothrix*, and *Nostoc* (Carmichael, 1992; Watanabe et al., 1992; Tillett et al., 2000; Keil et al., 2002). More than 200 different structural analogs of MCs have been identified from cyanobacterial blooms and cultures (Zastepa et al., 2015). Microcystin-LR is the most common and potent analog, followed by microcystin-RR and microcystin-YR (Chen et al., 2016). Many of the threats that cyanobacterial blooms pose to humans and animals are from MCs (Carmichael et al., 2001; Azevedo et al., 2002). These compounds are active in inhibition of protein phosphatase 1 and 2A and tumor promotion (Mackintosh et al., 1990; Campos and Vasconcelos, 2010), causing severe health risks to plants, animals, and humans. MCs are also structurally stable and resistant to many physical and chemical processes such as high temperature, sunlight, chemical hydrolysis, and oxidation (Wormer et al., 2010; Rastogi et al., 2014). However, some bacterial populations occurring in natural waters have been reported to degrade MCs effectively (Edwards et al., 2008; Dziga et al., 2013; Kormas and Lymperopoulou, 2013), providing a promising approach to elimination of MCs from natural waters. Many indigenous bacteria from lakes and reservoirs that can degrade MCs have been isolated to investigate MC degradation (Eleuterio and Batista, 2010; Jiang et al., 2011; Yang et al., 2014; Zhang et al., 2015; Zhu et al., 2016). Sphingomonadaceae bacteria have been focused on because many are effective in MC degradation (Valeria et al., 2006; Zhang et al., 2010; Xiao et al., 2011; Maghsoudi et al., 2016; Ding et al., 2018).

Typically, a cluster of four genes (*mlrA, mlrB, mlrC*, and *mlrD*) has been characterized as being responsible for MC degradation (Bourne et al., 1996, 2001). The *mlrA* gene encodes a microcystinase (MlrA), which can cleave the cyclic MC structure into a 2100-times less toxic linearized molecule by breaking it at the Arg–Adda peptide bond and is considered to be an important marker for the detection of MC-degrading bacteria (Bourne et al., 1996; Dziga et al., 2012). MlrB hydrolyzes the linearized MC-LR at Leu–Ala to form a tetrapeptide (Bourne et al., 1996). MlrC can decompose both linearized MC-LR and the tetrapeptide into Adda through the fracture of the Adda–Glu bond (Hashimoto et al., 2009; Shimizu et al., 2012; Dziga et al., 2016). MlrD was predicted to be an oligopeptide transporter because of its potential transmembrane spanning regions (Bourne et al., 2001). Apart from the *mlr* gene cluster, some alternative MC degradation pathways have been reported. Edwards et al. (2008) found demethylation, hydrolysis, decarboxylation, and condensation of microcystin LF (MC-LF), with nodularin as a novel intermediate degradation product. Dziga et al. (2017) detected four products of dmMC-LR in temperate freshwater bodies, including cyclic dmMC-LR, two cyclic dmMC-LRs with different modifications in the Arg–Asp–Leu region, and a tetrapeptide. Zhang et al. (2010) found that *Sphingopyxis* sp. USTB-05 can degrade MC-RR through hydrolysis and dehydration reactions to form a mostly linear MC-RR with two small peptide rings. A recent report identified some new MC-LR metabolites, such as Mdha–Ala, MeAsp–Arg, and Leu, indicating that there may be other novel types of hydrolases (Ding et al., 2018). More recently, a novel degradation pathway in which MC-LR is hydrolyzed by cleaving the Ala–Mdha peptide to a new linear MC-LR intermediate in anaerobic conditions was proposed (Zhu et al., 2019). Previous studies revealed that the glutathione-S-transferase (GST) and the CAAX-type II amino-terminal protease present in MC-degrading bacteria are involved in MC degradation (Ding et al., 2000; Saito et al., 2003; Gehringer et al., 2004; Maatouk et al., 2004; Dziga et al., 2013; Kansole and Lin, 2016). Analysis of whole genome gene expression may help identify further enzymes that are activated during MC degradation.

*Novosphingobium* sp. THN1, isolated from a water sample from Lake Taihu, China, belongs to the family Sphingomonadaceae (Jiang et al., 2011). This bacterial strain can degrade MC-LR effectively, eliminating 91.2% of MC-LR in THN1 culture during the first 12 h and completely removing MC-LR after 60 h. Like other microcystin-degrading Sphingomonadaceae, THN1 contains a *mlr* gene cluster that was confirmed to be involved in MC degradation (Bourne et al., 2001). The *mlrA* gene expression of THN1 during MC degradation was investigated (Jiang et al., 2011), and the results revealed that MC-LR induced upregulation of *mlrA*, with the highest transcription level occurring at 45 min.

Biodegradation of MCs in the environment has been widely studied. Environmental factors such as nutrient conditions (Li et al., 2011), oxygen status (Holst et al., 2003), temperature (Park et al., 2001; Chen et al., 2010), and MC-LR and chlorophyll *a* concentration (Li et al., 2015) in water are reported to affect the biodegradation rate of MCs. Glucose was used to investigate the effect of organic carbon on MC-LR degradation and contradictory effects have been noted in previous studies. Some studies showed that glucose added as exogenous carbon sources increased the removal percentage of MC-LR by *Lactobacillus plantarum* IS-10506, *Lactobacillus plantarum* IS-20506, and *Bacillus nanhaiencis* JZ-2013 in medium (Surono et al., 2008; Zhang et al., 2015), while others drew the opposite conclusion that addition of glucose to cultures repressed MC-LR degradation (Eleuterio and Batista, 2010; Li et al., 2011, 2012). Furthermore, *mlrA* gene expression and gene abundance were assessed during MC-LR biodegradation. Some previous studies of MC degradation by the microbial communities in environmental samples confirmed that the abundance of the *mlrA* gene in the community increased with MC-LR removal (Eleuterio and Batista, 2010; Ho et al., 2010; Li et al., 2012; Lezcano et al., 2016). A recent study showed that the variety and the number of MC-degrading microbes decreased dramatically on the addition of high concentrations of glucose (Ma et al., 2016). Divergent responses of functional gene expression to various nutrient conditions were revealed during MC-LR biodegradation by *Novosphingobium* sp. THN1. However, to the best of our knowledge, no previous work has assessed both *mlrA* gene expression and bacterial biomass during MC degradation. It is unclear whether bacterial populations, functional gene expression, or both, regulate MC degradation. The reported contradictory nutrient effects on MC-LR biodegradation may be due to differences in cell growth state or regulation of gene expression.

The aim of this study was to analyze the effect of carbon availability on the degradation rate of MC-LR by strain THN1 and explore the possible regulatory mechanism(s). We first assessed the MC-LR biodegradation rate at different carbon concentrations. Then, we linked the MC-LR biodegradation rate with *mlrA* gene expression and bacterial growth at different carbon concentrations. Significantly, we applied transcriptomics to identify the gene regulation mechanisms of MC-LR biodegradation at different carbon concentrations. Results from our transcriptomic data may help to clarify the whole-genome gene regulation of MC-LR biodegradation, and identify novel genes that are involved in MC-LR biodegradation.

## Materials and Methods

### Strain and Reagents

*Novosphingobium* sp. strain THN1 was obtained from the Laboratory of Harmful Algae Biology, Institute of Hydrobiology, Chinese Academy of Sciences. The strain was incubated in R2A medium (Table 1) at 37°C in the dark while shaking at 200 rpm.

**Table 1:** Concentrations of ingredients in the media used in this study.

Ingredient	100%C_R2A medium (g L^-1^)	70%C_R2A medium (g L^-1^)	40%C_R2A medium (g L^-1^)
Proteose Peptone	0.50	0.50	0.50
Starch	0.50	0.35	0.20
Glucose	0.50	0.35	0.20
Yeast Extract	0.50	0.50	0.50
Casein Hydrolysate	0.50	0.50	0.50
Dipotassium Phosphate	0.30	0.30	0.30
Sodium Pyruvate	0.30	0.21	0.12
Magnesium Sulfate Anhydrous	0.024	0.024	0.024

*The main carbon sources in R2A (100%C_R2A) medium are starch, glucose, and sodium pyruvate. The main carbon sources in R2A medium were decreased to 70 and 40%, respectively, in 70%C_R2A and 40%C_R2A medium.*

MC-LR (≥95% purity, Lot No.: L1101003, CAS No.: 101043-37-2) was purchased from Taiwan Algal Science Incorporation and stored at -20°C. Upon use, MC-LR was dissolved in methanol to prepare a stock solution.

### MC-LR Biodegradation and THN1 Growth at Different Carbon Concentrations

The ingredients of R2A medium are listed in Table 1. Glucose, starch, and sodium pyruvate are the main carbon sources. To investigate the effect of carbon availability on MC-LR biodegradation, we decreased the three main carbon ingredients of R2A medium to 70% and 40% (70%C_R2A and 40%C_R2A medium, respectively). The ingredients of the modified media are also listed in Table 1.

Exponentially growing cultures of THN1 were harvested and centrifuged at 6000× *g* for 10 min. Cell pellets were washed and resuspended at a final absorbance of 0.3 measured at 600 nm (OD_600_) in media with different organic carbon concentrations (100%C_R2A, 70%C_R2A, and 40%C_R2A). MC-LR was then spiked into the culture at a final concentration of 1.5 or 3 mg L^-1^. Autoclaved cultures were set as controls to account for any abiotic loss of MCs. All the groups were shaken at 37°C in the dark. In addition, R2A medium containing 1 or 5 mg L^-1^ MC-LR was prepared to examine the growth of THN1 in different concentrations of MC-LR; bacterial cultures spiked with an equivalent amount of sterile ultrapure water were used as controls.

At reaction times of 0, 1, 2, and 3 h, samples were taken and centrifuged at 6000× *g* for 10 min. The supernatants were collected for MC-LR analysis while the precipitates were used for RNA extraction. Moreover, 3-mL samples were collected and monitored for bacterial growth by measuring OD_600_ values. The experiments were conducted in triplicate and the average values were used for analysis.

### MC-LR Analysis

Cell cultures collected at 0, 1, 2, and 3 h were centrifuged at 6000× *g* for 10 min. The supernatant was collected and filtered through 0.22-μm syringe filters (PTEE Hydrophilic, Millipore, United States) before analysis. The concentration of MC-LR (standard) for preparing the calibration curve ranged between 0.1 and 5 μg L^-1^.

The MC-LR concentration in the supernatant was determined using an enzyme-linked immunosorbent assay kit (IHB, CAS, China) according to Lei’s method (Lei et al., 2004). Briefly, samples were diluted based on predicted MC-LR concentration, so that the final MC-LR concentration in each sample was theoretically within the range of standards supplied. Anti-MC-LR monoclonal antibodies were mixed with MC-LR standards or diluted samples in microtiter plates (Nunc, Denmark). After incubation at 37°C for 1 h, the plates were washed, and europium-labeled antimouse IgG conjugate (Perkin-Elmer), diluted 1:500 in assay buffer (Perkin-Elmer), was added at 100 μL/well. After a further incubation for 1 h at 37°C, the plates were washed six times, and enhancement solution (Perkin-Elmer) was added at 100 μL/well. The plates underwent rotation incubation for 5 min and then the concentration of MC-LR was measured at 450 nm using a Microplate Spectrophotometer Reader (Thermo Scientific^TM^ Multiskan^TM^ GO Microplate Spectrophotometer type 357, Thermo Scientific, Helsinki, Finland).

The average biodegradation percentage was calculated by dividing the concentration of MCs initially spiked into the cultures by the remaining MC concentration in the sample. The biodegradation rate was the average biodegradation percentage per h.

### RNA Extraction and Reverse Transcription

Bacterial RNA from *Novosphingobium* sp. THN1 was extracted using an E.Z.N.A. Bacterial RNA kit (Omega) according to the manufacturer’s protocol. The amount and purity of the extracted RNA were determined using comparison of the optical density at 260 and 280 nm by spectrophotometry (NanoDrop 8000, Thermo Fisher Inc., United States) and agarose gel electrophoresis to evaluate integrity (Rastogi et al., 2014). Samples were then stored at -80°C. After digestion with DNase I (Promega), 2 μg of total RNA was reverse transcribed using a RevertAid first-strand cDNA synthesis kit (Thermo Fisher Scientific, Waltham, MA, United States) according to the kit manual.

### Real-Time qPCR Analyses

Real-time qPCR reactions were performed on the Roche LightCycler 480 Real-Time PCR system (Roche, United States) using cDNA (see section “RNA Extraction and Reverse Transcription”). Two pairs of specific primers, qmlrAF/qmlrAR, and q16SF/q16SR, were used to quantify the number of copies of the *mlrA* and 16S ribosomal ribonucleic acid (rRNA) genes, respectively (Table 2). The 16S rRNA gene acted as the housekeeping gene in qPCR assays. Real-time qPCR assay and analysis were performed as described previously (Li et al., 2014). All reactions were completed in a total volume of 20 μL, containing 10 μL Master Mix (SYBR Green, Toyobo, Japan), 1 μL (10 μmol L^-1^) of each primer, 1 μL cDNA template, and ddH_2_O. The qRT-PCR program was: 95°C for 4 min, followed by 40 cycles of 95°C for 15 s, 60°C for 30 s, and 72°C for 45 s. Gene expression results were assessed through the Ct value. The relative expression ratio (Rastogi et al., 2014) was calculated by formula 2^-ΔΔCt^ based on the equation: ΔΔCt = (C_t, target gene_ – C_t,16SrRNA_)_stress_ – (C_t, target gene_ – C_t,16SrRNA_)_control_. All assays were performed in triplicate and the results are reported as means [±standard deviation (SD)].

**Table 2:** Primers used in this study.

Target gene	Primer	Sequence (5′-3′)	References
*16SrRNA*	q16SF	CGTAAAGCTCTTTTGCCAGGGA	Jiang et al. (2011)
	q16SR	CTTTCACCTCTGACTTGTGTCGC	
*mlrA*	qmlrAF	AGGAGACGCACGCTCACCTC	Jiang et al. (2011)
	qmlrAR	GGCTATGACAGTAACGCCCTGA	

### Transcriptome Sequencing and Analysis

RNA samples extracted from cells spiked with MC-LR at 0 and 2 h in 100%C_R2A and 40%C_R2A media were used for transcriptome sequencing. The mRNA was sheared into fragments in fragmentation buffer, and used for first-strand cDNA synthesis using reverse transcriptase and random primers. This was followed by second strand cDNA synthesis using DNA polymerase I and RNase H. These cDNA fragments were purified with a QiaQuick PCR extraction kit, and underwent end repair and ligation of adapters. The products were purified, and fragments with an approximate size of 350 bp were selected by agarose gel-electrophoresis. Sequencing libraries were constructed by amplifying the selected fragments by PCR. The libraries were sequenced using the Illumina HiSeq 2000 platform, and raw reads were generated. Qualified sequences were mapped to the *Novosphingobium* sp. THN1 genome (Wang et al., 2018) using Bowtie2 (Coronado et al., 2012) with no more than five mismatched bases. The datasets generated for this study can be found in NCBI Gene Expression Omnibus, GSE125827.

### Analysis of Differentially Expressed Genes (DEGs)

To compare the differences in gene expression between the control (0 h) and MC-LR-treated samples, mRNA abundance was estimated by the number of uniquely mapped reads per kilobase per million reads (RPKM) method (Bullard et al., 2010). The calculated gene expression was used to compare the differentially expressed genes (DEGs) between samples. A false discovery rate (FDR) control was used to obtain the true number of DEGs (Audic and Claverie, 1997). Differential expression between the treatment and reference conditions was computed with DESeq (Anders and Huber, 2010). The DEGs were defined as those with an FDR < 0.001 and an RPKM ratio of the two samples |log_2_ ratio|≥ 1. The fold-changes of DEGs were calculated as the log_2_ ratios of the gene abundance comparing MC-LR treated-samples and the control sample.

The biological functions of the DEGs were identified to investigate the pattern of transcriptome regulation that occurred during MC-LR biodegradation at different carbon concentrations. Clusters of Orthologous Groups (COG) annotations of the DEGs were used for functional classification. Kyoto Encyclopedia of Genes and Genomes (KEGG) pathway annotations were performed^1^ to determine the cellular pathways involving the DEGs.

## Results

### MC-LR Degradation by THN1 at Different Carbon Concentrations

**FIGURE 1:**
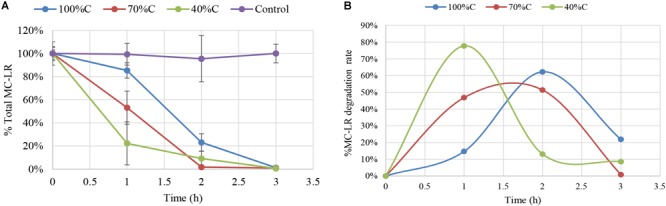
Biodegradation of total microcystin (MC)-LR by *Novosphingobium* sp. strain THN1 incubated at different carbon concentrations. **(A)** The remaining MC-LR percentage after 3 h; **(B)** The biodegradation percentage of MC-LR over 3 h. The initial MC-LR concentration was 1.5 mg L^-1^.

Figure 1 shows degradation kinetics of 1.5 mg L^-1^ MC-LR by *Novosphingobium* sp. strain THN1 at different concentrations of carbon sources. No obvious decrease in the initial MC-LR concentration was observed in controls, indicating that any loss of MC-LR in the experimental groups was attributable to biodegradation.

At all carbon concentrations, MC-LR concentration declined dramatically in the first 3 h and >95% of MC-LR was degraded. The MC-LR degradation rate peaked early and then declined. MC-LR biodegradation by THN1 was different at different carbon concentrations. In comparison with the MC-LR biodegradation in R2A medium, decreased carbon concentration caused significant stimulation of MC-LR biodegradation at 1 h: the remaining MC-LR percentage under sequentially decreased carbon concentrations (100%C_R2A, 70%C_R2A, and 40%C_R2A) was 85.31 ± 6.65%, 53.17 ± 14.32%, and 22.26 ± 18.58%, respectively (Figure 1A). Correspondingly, the MC-LR degradation rate increased as the carbon level decreased. The biodegradation percentage of MC-LR at 2 and 3 h were different from that of 1 h. At 2 h of MC-LR degradation, the biodegradation rate decreased as the carbon concentration decreased. The biodegradation rate at 3 h in 40%C_R2A medium was lower than in 100%C_R2A medium and higher than in 70%C_R2A medium. As Figure 1B shows, the degradation peaks occurred at 1, 1.5, and 2 h for 40%C_R2A, 70%C_R2A, and 100%C_R2A, respectively. Decreased carbon stimulates the advance of the degradation peak.

### THN1 Growth During MC-LR Degradation

**FIGURE 2:**
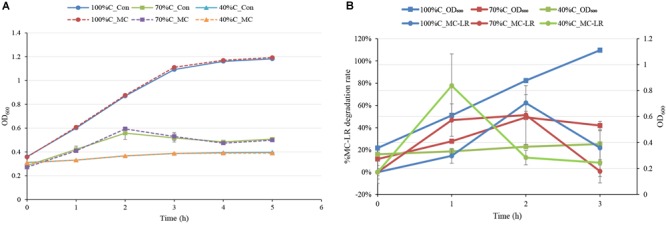
**(A)** The absorbance of THN1 cultures at 600 nm at different carbon concentrations during MC-LR biodegradation. **(B)** Kinetic relationship between MC-LR removal and THN1 growth at different carbon concentrations.

The MC-degrading bacterium THN1 did not exhibit significant differences in growth at any carbon concentration when 1.5 mg L^-1^ MC-LR was added. The treatment cultures and controls reached similar OD_600_ values in the first 5 h (Figure 2A). As expected, the growth of THN1 in decreased carbon concentrations was slower than that in normal R2A medium. This result was consistent with previous observations on growth of MC-degrading bacteria that the presence of exogenous C and/or N stimulate the growth of such bacteria during MC-degradation (Zhang et al., 2015; Lezcano et al., 2016). The lower the carbon concentration, the slower the growth and thus the lower the cell population. Meanwhile, the OD_600_ of THN1 declined during the monitoring period in control medium without carbon sources, indicating that THN1 cannot grow in inorganic medium lacking a carbon source.

As previous observation showed that the abundance of the *mlrA* gene responded positively to the release of MCs in waters (Zhu et al., 2014; Li et al., 2015; Lezcano et al., 2016), we assessed the growth of THN1 at different concentrations of MC-LR (1, 1.5, and 5 mg L^-1^). There was no significant difference in the cell growth of cultures treated with MC-LR and controls (data not shown). These findings suggest that MC-LR cannot promote THN1 growth. Figure 2B showed that THN1 growth was not correlated with MC-LR degradation rate. The increased *mlrA* gene abundance in response to MCs in waters remained to be explored.

### *mlrA* Gene Expression During MC-LR Degradation

**FIGURE 3:**
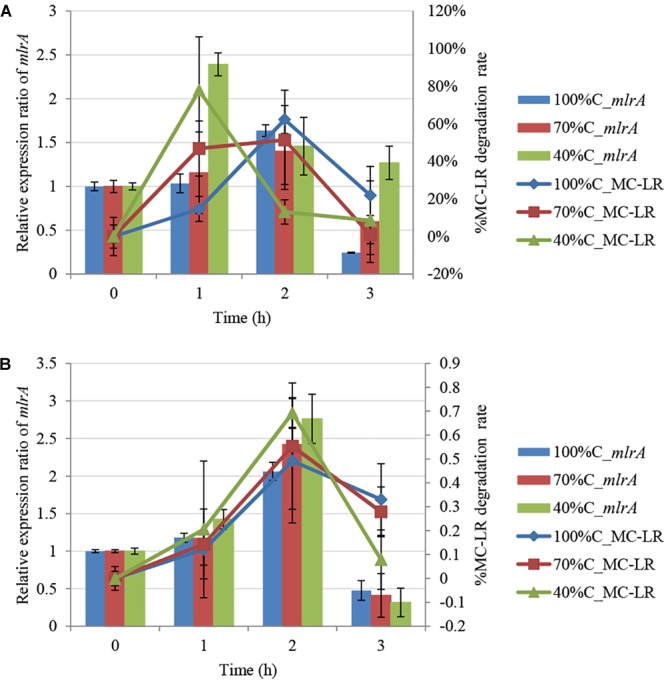
Kinetic relationship between MC-LR removal and *mlrA* gene expression at different carbon concentrations: **(A)** 1.5 mg L^-1^ MC-LR; **(B)** 3 mg L^-1^ MC-LR.

Figure 3 shows *mlrA* gene expression in THN1 cells in the first 3 h of MC-LR biodegradation at different carbon concentrations. After addition of MC-LR, expression of *mlrA* increased immediately and subsequently declined at all carbon concentrations. Exposure to MC-LR induced upregulation of *mlrA* expression in the first hour at all carbon concentrations. This indicates that MC-LR promotes the activity of MC-degrading enzymes. Consistently, Shimizu et al. (2011) revealed that the addition of MCs increased the MC degradation rate and *mlrA* gene expression of *Sphingopyxis* sp. C-1 in 30 min.

Furthermore, we found that as the carbon concentration declined, the relative expression ratio of *mlrA* increased at 1 h and decreased at 3 h. At 1 h, the ratio of *mlrA* gene expression compared with the level of *mlrA* in the absence of MC-LR at sequentially decreased carbon concentrations (100%C_R2A, 70%C_R2A, and 40%C_R2A) was 1.034 ± 0.107, 1.156 ± 0.465, and 2.392 ± 0.131, respectively, i.e., lower carbon concentrations induced more *mlrA* gene expression. The relative expression ratio of *mlrA* gene at 3 h at sequentially decreased carbon concentrations (100%C_R2A, 70%C_R2A, and 40%C_R2A) was 0.2445 ± 0.007, 0.598 ± 0.466, and 1.2685 ± 0.19, respectively. We suspect that a decrease of carbon concentration induced sensitive *mlrA* gene expression; the upregulation peak of *mlrA* was earlier and the subsequent decrease slower at lower carbon concentrations.

### *mlrA* Gene Regulation of MC-LR Degradation by THN1

During MC-LR biodegradation by THN1, there was no positive correlation between the bacterial biomass and MC-LR degradation at different carbon concentrations. However, *mlrA* gene expression showed a similar trend to the degradation of MC-LR. At 1 h, at decreased carbon concentration, *mlrA* gene expression was upregulated relative to that of 0 h and the MC-LR degradation percentage increased. At 2 h, the expression ratio of *mlrA* gene in 70%C_R2A medium was a little less than that in 40%C_R2A medium, which mirrored exactly the MC-LR degradation percentage at the two carbon concentrations. The removal trend of MC-LR followed closely the trend of *mlrA* gene expression during 1–3 h at each carbon concentration (Figure 3). There was a positive correlation between the MC-LR degradation percentage and the expression ratios of *mlrA* at different organic carbon concentrations (100%C_R2A, 70%C_R2A, and 40%C_R2A) (r^2^ = 0.717, *p* < 0.05), while the biomass was not related to the degradation rate. This correlation was also found in the degradation of 3 mg L^-1^ MC-LR (Figure 3B). Thus, we suspect that MC-LR biodegradation may be gene regulated rather than biomass regulated; MC-LR use may lead to an increase in the expression of bacterial MC-LR biodegradation-related genes and the upregulation of *mlr* gene expression helps bacteria degrade more MC-LR.

### Whole-Genome Expression Analysis

**FIGURE 4:**
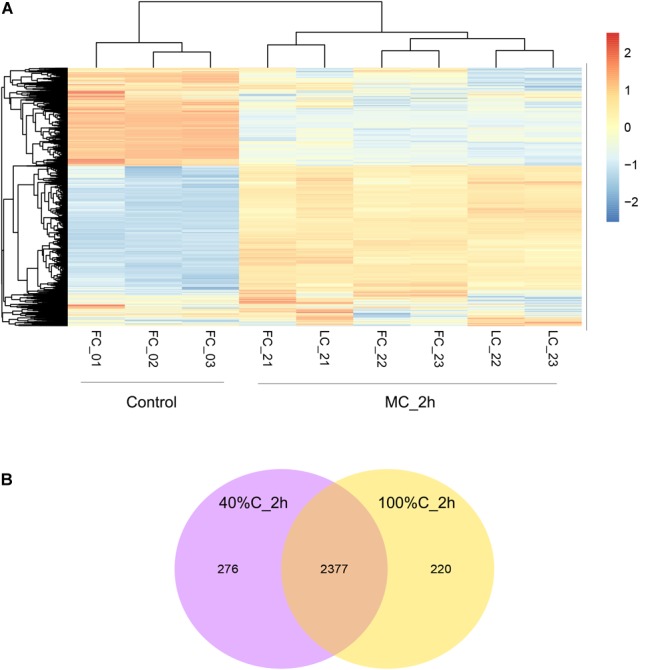
**(A)** Hierarchical clustering of THN1 gene expression on exposure to MC-LR at 100%C_R2A and 40%C_R2A at 0 and 2 h. FC_0^∗^ refers to transcriptional profiles at 0 h in 100%C_R2A medium, FC_2^∗^ to that at 2 h in 100%C_R2A medium, and LC_2^∗^ refers to transcriptional profiles at 2 h in 40%C_R2A medium. **(B)** Venn diagram grouping the genes differentially expressed on exposure to MC-LR at 2 h compared to 0 h.

**FIGURE 5:**
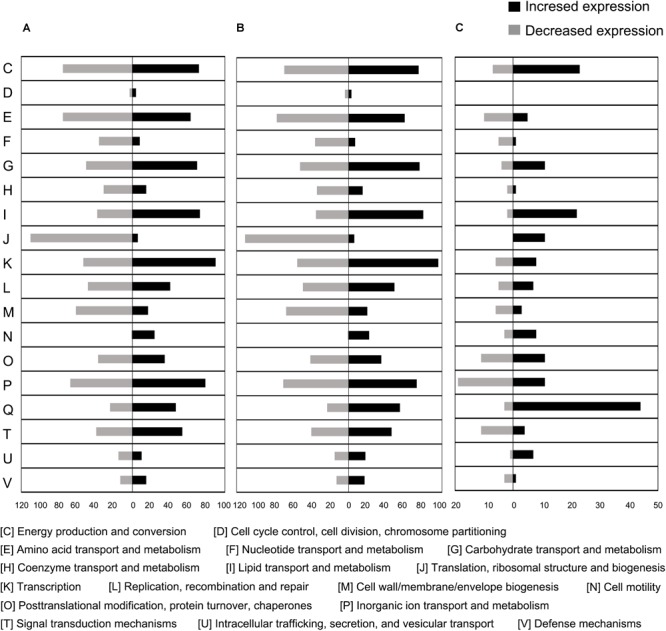
Proportional abundance of differentially expressed genes in conditions of MC-LR exposure, categorized by Clusters of Orthologous Genes (COG). **(A)** 100%C_R2A at 2 h compared to 0 h, **(B)** 40%C_R2A at 2 h compared to 0 h, **(C)** 100%C_R2A at 2 h compared to 40%C_R2A at 2 h.

To further explore gene expression during MC-LR degradation, we used RNA-Seq analyses to examine genome-wide gene expression in strain THN1 at 2 h of MC-LR degradation in 100%C_R2A and 40%C_R2A carbon conditions. At 2 h, DEGs shared similar gene expression patterns in 100%C_R2A and 40%C_R2A carbon conditions (Figure 4A). In comparison with 0 h, 62.16% (2597/4178) and 63.50% (2653/4178) of genes were differentially expressed at 2 h of MC-LR degradation at 100%C_R2A and 40%C_R2A, respectively. A total of 2377 genes were commonly differentially expressed at both carbon concentrations, indicating a large proportion of DEGs induced by MC-LR (Figure 4B). Among the common DEGs, 47.50% were upregulated and 52.2% were downregulated after 2 h of MC-LR degradation compared to 0 h. Globally speaking, the COG distribution of DEGs at 100%C_R2A and 40%C_R2A during MC-LR degradation was similar (Figure 5). During MC-LR degradation, a large proportion of DEGs were grouped in categories COG-C (*Energy production and conversion*), COG-E (*Amino acid metabolism*), COG-G (*Carbohydrate transport and metabolism*), COG-I (*Lipid metabolism*), COG-J (*Translation, ribosomal structure and biogenesis*), and COG-K (*Transcription*). At 2 h, in comparison to 100%C_R2A carbon conditions, many upregulated genes at 40%C_R2A grouped in COG-C (*Energy production and conversion*), COG-I (*Lipid metabolism*), and COG-Q (*Secondary metabolites biosynthesis, transport and catabolism*).

**FIGURE 6:**
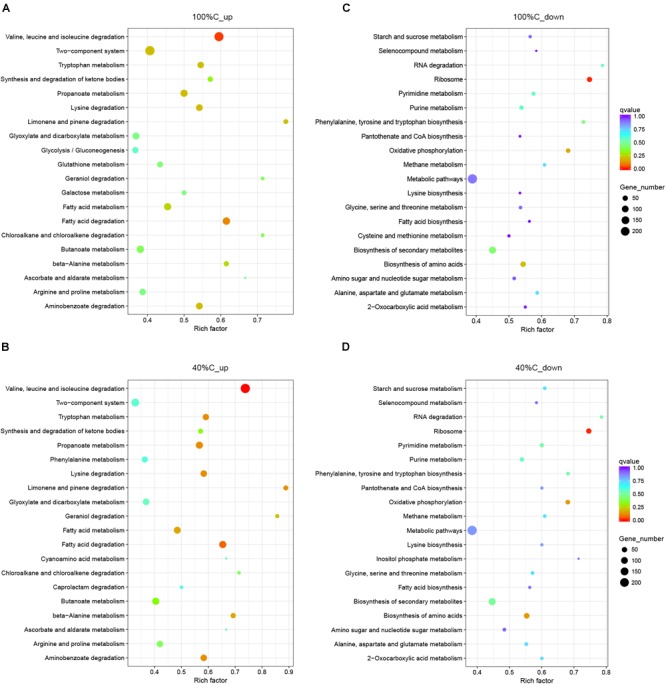
Functional categories of upregulated **(A,B)** and downregulated genes **(C,D)** on exposure to MC-LR at 100%C_R2A and 40%C_R2A, respectively. The size of the circles represent the total number of genes that are assigned to each functional category. The rich factor represents the ratio between enriched gene numbers and annotated gene numbers. The Q-value represents the corrected *p*-value. If the rich factor is larger and the Q-value is closer to 0, the enrichment is more significant. Functional categories with the top 20 enrichments are shown.

**FIGURE 7:**
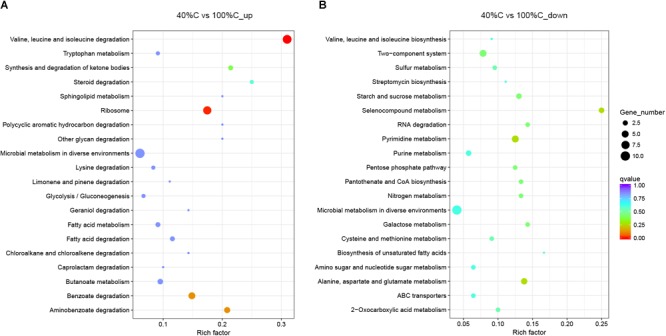
Functional categories of upregulated **(A)** and downregulated genes **(B)** after 2 h of exposure to MC-LR in 40%C_R2A compared to that in 100%C_R2A. The size of the circles represent the total number of genes that are assigned to each functional category. The rich factor represents the ratio between enriched gene numbers and annotated gene numbers. The Q-value represents the corrected *p*-value. If the rich factor is larger and the Q-value is closer to 0, the enrichment is more significant. Functional categories with the top 20 enrichments are shown.

Kyoto Encyclopedia of Genes and Genomes pathway analysis indicated that the upregulated DEGs during MC-LR degradation were mainly related to amino acid degradation and substrate metabolism pathways, such as valine, leucine and isoleucine degradation, tryptophan metabolism, butanoate metabolism, arginine and proline metabolism, and aminobenzoate degradation. Downregulated DEGs during MC-LR degradation were involved in the ribosome, biosynthesis of amino acids, oxidative phosphorylation, and biosynthesis of secondary metabolites (Figure 6). The expression of DEGs increased at 40%C_R2A compared to 100%C_R2A (Figure 7) including genes for valine, leucine and isoleucine degradation, the ribosome, benzoate degradation, and aminobenzoate degradation, while those that decreased included genes for pyrimidine metabolism, purine metabolism, the pentose phosphate pathway, nitrogen metabolism, alanine, aspartate, and glutamate metabolism. This suggests that MC-LR degradation differentially regulated a large number of genes involved in carbon, nitrogen, and amino acid metabolism. During MC-LR degradation, decreased carbon concentration also upregulated carbon- and nitrogen-related pathways.

### Downregulation of Carbon- and Nitrogen-Related Pathways

A considerable number of DEGs during MC-LR degradation encoded proteins related to carbon-, nitrogen- and amino acid-related pathways. At 2 h of MC-LR degradation, most DEGs (29/33) involved in carbon metabolism were downregulated (Table 3). Decreased carbon concentration increased the fold-downregulation. MC-LR degradation upregulated *ncd2/pcd*, which encode nitronate monooxygenase, and *gdhA*, which encodes glutamate dehydrogenase (NADP^+^), while downregulating other genes involved in nitrogen metabolism (Table 4). Decreased carbon concentration also increased the fold-downregulation of nitrogen metabolism-related genes. Furthermore, at 2 h of MC-LR degradation compared with the gene expression of 0 h, we detected increased expression of glutathione (GSH) metabolism-related genes from transcriptomic data, such as GST family protein, glutathione peroxidase, S-(hydroxymethyl) glutathione dehydrogenase, and glutathione-dependent disulfide-bond oxidoreductase. Previous studies revealed that the glutathione pathway was involved in MC degradation (Gehringer et al., 2004; Maatouk et al., 2004; Dziga et al., 2013). We thus suspect that these degradative enzymes may act here in MC degradation. More studies are expected to reveal the functions of these upregulated genes.

**Table 3:** Genes involved in carbon metabolism and their differential expression in 100%C_R2A and 40%C_R2A after 2 h of exposure to MC-LR relative to that at 0 h.

Pathway	Protein	Gene name	Gene ID	100%C_R2A	40%C_R2A
Calvin-Benson-Bassham cycle	phosphoglycerate kinase	*pgk*	C7W88_RS13175	–1.8881	–2.297
	triosephosphate isomerase	*tpiA*	C7W88_RS12520	–1.5082	–1.6432
glycolysis/gluconeogenesis	glucokinase	*glk*	C7W88_RS12365	–2.3853	–3.1357
	phosphoglucomutase	*pgm*	C7W88_RS11010	–0.78001	–1.1774
	glucose-6-phosphate isomerase	*pgi*	C7W88_RS11855	–0.87915	–1.302
	triosephosphate isomerase	*tpiA*	C7W88_RS12520	–1.5082	–1.6432
	phosphoglycerate kinase	*pgk*	C7W88_RS13175	–1.8881	–2.297
	phosphoglycerate mutase	*gpmA*	C7W88_RS04095	–2.1473	–2.1183
	enolase	*eno*	C7W88_RS14090	–1.7147	–2.1119
	pyruvate dehydrogenase E2 component	*aceF*/*pdhC*	C7W88_RS19345	1.2114	1.386
	alcohol dehydrogenase (cytochrome c)	*exaA*	C7W88_RS07670	2.3517	3.1069
	isocitrate dehydrogenase (NADP)	*icd*	C7W88_RS09015	–1.7695	–1.8075
	2-oxoglutarate dehydrogenase E2 component	*sucB*	C7W88_RS10070	–0.48608	–0.45975
	succinate dehydrogenase, subunit B	*sdhB*	C7W88_RS14600	–0.60111	–0.94252
	succinate dehydrogenase, subunit A	*sdhA*	C7W88_RS11555	–0.81581	–0.79219
	fumarase, class I	*fumA/fumB*	C7W88_RS05170	–0.82806	–1.0099
pentose phosphate pathway	phosphogluconate dehydratase	*edd*	C7W88_RS12360	–1.9651	–2.6625
	2-dehydro-3-deoxyphosphogluconate aldolase	*eda*	C7W88_RS19935	1.046	0.8111
	glucose-6-phosphate isomerase	*pgi*	C7W88_RS11855	–0.87915	–1.302
	ribose-phosphate pyrophosphokinase	*prsA*	C7W88_RS01250	–2.4087	–2.743
	phosphoglucomutase	*pgm*	C7W88_RS11010	–0.78001	–1.1774
	6-phosphogluconolactonase	*devB*	C7W88_RS12355	–2.1998	–2.9099
amino sugar and nucleotide sugar metabolism	UDP-N-acetyl-D-mannosaminuronic acid dehydrogenase	*wecC*	C7W88_RS02750	–0.52148	–0.51175
	phosphoglucosamine mutase	*glmM*	C7W88_RS03760	0.61432	0.51499
	fructokinase	*scrK*	C7W88_RS12310	–2.1873	–1.8955
	UDP-N-acetyl-D-galactosamine dehydrogenase	*wbpO*	C7W88_RS02740	–0.94907	–1.1678
	glucose-6-phosphate isomerase	*pgi*	C7W88_RS11855	–0.87915	–1.302
	UDPglucose 6-dehydrogenase	*ugd*	C7W88_RS04045	–0.71709	–0.93493
	UDPglucose 6-dehydrogenase	*ugd*	C7W88_RS04045	–0.71709	–0.93493
	phosphoglucomutase	*pgm*	C7W88_RS11010	–0.78001	–1.1774
	glucokinase	*glk*	C7W88_RS12365	–2.3853	–3.1357
	mannose-6-phosphate isomerase	*manA*	C7W88_RS03390	–2.4986	–2.8024
	UDP-glucuronate 4-epimerase		C7W88_RS02735	–0.79956	–1.2682
	phosphomannomutase	*manB*	C7W88_RS17380	–1.8684	–0.59501
	mannose-1-phosphate guanylyltransferase	*manC*	C7W88_RS03395	–1.9549	–2.1469

**Table 4:** Genes involved in nitrogen metabolism and their differential expression in 100%C_R2A and 40%C_R2A after 2 h of exposure to MC-LR relative to that at 0 h.

Protein	Gene name	Gene ID	100%C_R2A	40%C_R2A
nitronate monooxygenase	*ncd2/npd*	C7W88_RS02470	1.456	2.1839
ammonium transporter	*amt*	C7W88_RS14530	–1.1864	–1.2739
ferric uptake regulator	*fur*	C7W88_RS01190	–0.52916	–0.64454
glutamate dehydrogenase (NADP^+^)	*gdhA*	C7W88_RS08975	2.9442	2.6938
glutamate synthase (NADPH) large chain	*gltB*	C7W88_RS03905	–1.7876	–1.9091
glutamate synthase (NADPH) small chain	*gltD*	C7W88_RS03900	–1.5872	–1.7881
L-aspartate oxidase	*nadB*	C7W88_RS06620	–0.50332	–0.68309

## Discussion

### Carbon Inhibition of MC-LR Biodegradation

Biodegradation is an ecofriendly and effective strategy for MC removal, and has been applied practically in water purification processes (Li et al., 2011, 2015; Huisman et al., 2018). However, in natural environments, MC-LR biodegradation can be inhibited by high variability of environmental factors such as temperature, bacterial composition, pH, and the presence of exogenous nutrients (Li et al., 2017; Lezcano et al., 2018). Carbon concentration is a main factor affecting the MC-LR biodegradation rate. Contradictory effects of glucose have been noted in previous studies. Park et al. (2001) reported that *Sphingomonas* sp. Y2 could use MCs as a carbon source. In the presence of alternative organic carbon sources, MC degradation was much slower than that in organic-free medium. Holst et al. (2003) observed a stimulatory effect on MC-LR degradation by glucose addition in anoxic conditions, and stimulation of MC degradation activity by glucose. Some other studies have also shown that MC-degrading bacteria may preferentially use glucose over MCs as a carbon source. We speculate that in our study, a preference of *Novosphingobium* sp. strain THN1 for the carbon sources in the medium over MC-LR resulted in the inhibition of MC-LR biodegradation at high carbon concentrations. However, this explanation does not apply to the results of Li et al. (2011), who reported a stimulatory effect of the addition of glucose on MC-LR biodegradation by biofilm. In their study, enhanced inhibition of MC-LR degradation was correlated with increased proliferation of MC-degrading bacteria as the concentration of nutrients increased. Varied strain characteristics were probably attributable to the presence of different species or unique functional genes.

Cao et al. (2018) investigated the effect of different types of organic matter on MC-LR degradation in soils. They observed that MC-degradation was stimulated by the addition of humic acid but inhibited by the addition of glucose and glycine. Our study may provide advice for regulating optimal carbon concentrations to biodegrade MC-LR efficiently. In the field scale, we may decrease the carbon concentrations by throwing heterotrophic bacteria into the lake or pond in order to reduce the inhibition of high carbon concentrations on MC-LR degradation.

### MC-LR Biodegradation Was Related to *mlrA* Gene Expression Rather Than Biomass

The kinetics of MC-biodegradation and *mlrA* gene abundance have been evaluated in various nutrient conditions. Expression profiles of the *mlrA* gene were detected during MC-LR degradation by MC-degrading *Sphingopyxis* sp. m6 isolated from Lake Taihu (Ding et al., 2018). In that study, *mlrA* gene expression showed a rapid increase in the first hour and then a gradual decline to the control level from 2 to 6 h. That result was consistent with our observations. Previously, a correlation between proteolytic activity and MC removal was found for *Lactobacillus rhamnosus* GG, *Lactobacillus rhamnosus* LC-705, and *Bifidobacteriu longum* 46 and both these parameters were dependent on glucose as an energy source (Nybom et al., 2012). These reports combined with our results may suggest that MC degradation mainly results from the expression of functional *mlrA* genes. The question of why different concentrations of organic carbon affected *mlrA* gene expression the way they did remains to be explored.

### MC-LR Did Not Promote Growth of *Novosphingobium* sp. THN1

Microcystin biodegradation kinetics of several isolated bacterial populations have been quantified. Zhang et al. (2015) revealed that when 10 mg/L of glucose and ammonium chloride were added as exogenous carbon and nitrogen sources, the degradation percentage of MC-LR by *Bacillus nanhaiencis* JZ-2013 increased from about 80% to about 90%. At the same time, the bacterial growth also improved. When combined with our results, one may conclude that the improved bacterial growth resulted from the added carbon or nitrogen sources, but not from MCs. However, Valeria et al. (2006) observed that MC-RR biodegradation by *Sphingomonas* sp. CBA4 was accompanied by a slight increase in bacterial density. Also, *Sphingopyxis* sp. m6 maintained moderate growth in the first 3 h of MC-LR degradation and then rapidly increased after MC-LR was decomposed (Ding et al., 2018). However, in our study, the presence or absence of MC-LR did not result in a significant difference in bacterial growth. MC-LR cannot be assimilated as a carbon source for THN1 growth. Recently, a comparison of several unstructured kinetic models to describe MC biodegradation by isolated degrading populations was reported (Manheim et al., 2019). Model predictions suggest that MC concentrations in the environment are well below saturating levels for optimal bacterial growth. Thus, MC degradation may fail in promoting bacterial growth. Previously, two studies reported that during MC-LR degradation, the degrading bacteria did not use MC-LR as a carbon source and MC-LR might be removed via a xenobiotic mechanism. As a result, the total bacterial concentration was not increased by MC-LR (Mou et al., 2013; Kansole and Lin, 2016). Xenobiotic MC-LR degradation may also explain why MC-LR degradation did not lead to an increase in bacterial growth in the present study.

### MC-LR Degradation Upregulated Other Degradative Genes

Alternative microcystin enzymatic degradation pathways apart from the *mlr* gene cluster have been reported (Dziga et al., 2013). CAAX type II amino-terminal protease belonging to the CAAX Proteases and Bacteriocin Processing Enzymes family might encode a microcystinase function (Read et al., 2002; Pei et al., 2011) and be involved in MC-LR degradation (Kansole and Lin, 2016). Mou et al. (2013) reported over-representation of GST and cytochrome P450 oxidase during MC-LR degradation, which are proposed to catalyze the synthetic metabolism of MC-LR to cysteine and GSH conjugates in animals (Campos and Vasconcelos, 2010). Several studies also found some MC-degrading bacteria that did not contain *mlr* genes (Manage et al., 2009; Yang et al., 2014; Zhu et al., 2019). Also, here, we detected increased expression of GSH metabolism-related genes in our transcriptomic data (GST family protein, glutathione peroxidase, S-[hydroxymethyl] glutathione dehydrogenase, and glutathione-dependent disulfide-bond oxidoreductase). As the transcriptomics analysis in this study showed that MC-LR degradation upregulated degradative genes aside from *mlr*, these degradative genes may be involved in MC-LR degradation by bacteria and account for the production of novel metabolites. More studies should thus be performed to explore the functions of these degradative genes during MC-LR degradation in the natural environment.

Microcystin removal efficiency was also dependent on temperature, pH, and cell density when glucose was added to the medium (Nybom et al., 2008). Determination of the regulatory mechanisms of different environment factors will better clarify MC-LR degradation regulation in practice.

## Conclusion

We decreased the carbon concentration of R2A medium to assess MC-LR degradation by the MC-degrading bacterium *Novosphingobium* sp. THN1 and explored the possible regulatory mechanisms. MC-LR degradation peaked early during MC-LR biodegradation and then declined. Decreases in the carbon level stimulated the advance of the degradation peak. The *mlrA* expression level correlated with the MC-LR degradation rate by strain THN1. During MC-LR degradation, most genes involved in the ribosome, biosynthesis of amino acids, nitrogen biosynthesis, starch and sucrose metabolism, and biosynthesis of secondary metabolites were downregulated. This indicated that MC-LR regulates carbon and nitrogen pathways of *Novosphingobium* sp. THN1. Genes involved in amino acid degradation and substrate metabolism pathways were upregulated during MC-LR degradation.

## Author Contributions

TL and JZ conceived the study and designed the experiments. JW performed the experiments, carried out the data analysis, and prepared the first draft of the manuscript. CW and JL assisted in the formatting of the figures. QL and MS assisted in the data analysis. PB and YL helped to revise the manuscript. NG assisted in determining the MC-LR concentrations. All authors participated in the discussion of the manuscript, agreed to the final content, and read and approved the final manuscript.

## Conflict of Interest Statement

The authors declare that the research was conducted in the absence of any commercial or financial relationships that could be construed as a potential conflict of interest.

## Footnotes

1 http://www.genome.jp/kegg/
